# The honeybee (*Apis mellifera*) developmental state shapes the genetic composition of the deformed wing virus-A quasispecies during serial transmission

**DOI:** 10.1038/s41598-020-62673-w

**Published:** 2020-04-06

**Authors:** Orlando Yañez, Julio Chávez-Galarza, Christian Tellgren-Roth, M. Alice Pinto, Peter Neumann, Joachim R. de Miranda

**Affiliations:** 1 Institute of Bee Health, Vetsuisse Faculty, University of Bern, Bern, CH-3000 Switzerland; 2 Centro de Investigação de Montanha (CIMO), Instituto Politécnico de Bragança, Campus de Sta. Apolónia, 5300-253 Bragança, Portugal; 3 Instituto Nacional de Innovación Agraria (INIA), Av. La Molina, 1981 Lima, Perú; 4 SciLife Lab, BioMedical Centre, Uppsala University, Uppsala, 751 08 Sweden; 5 Department of Ecology, Swedish University of Agricultural Sciences, Uppsala, 750 07 Sweden

**Keywords:** Viral evolution, Virus-host interactions

## Abstract

The main biological threat to the western honeybee (*Apis mellifera*) is the parasitic mite *Varroa destructor*, largely because it vectors lethal epidemics of honeybee viruses that, in the absence of this mite, are relatively innocuous. The severe pathology is a direct consequence of excessive virus titres caused by this novel transmission route. However, little is known about how the virus adapts genetically during transmission and whether this influences the pathology. Here, we show that upon injection into honeybee pupae, the deformed wing virus type-A (DWV-A) quasispecies undergoes a rapid, extensive expansion of its sequence space, followed by strong negative selection towards a uniform, common shape by the time the pupae have completed their development, with no difference between symptomatic and asymptomatic adults in either DWV titre or genetic composition. This suggests that the physiological and molecular environment during pupal development has a strong, conservative influence on shaping the DWV-A quasispecies in emerging adults. There was furthermore no evidence of any progressive adaptation of the DWV-A quasispecies to serial intra-abdominal injection, simulating mite transmission, despite the generation of ample variation immediately following each transmission, suggesting that the virus either had already adapted to transmission by injection, or was unaffected by it.

## Introduction

The requirement of viruses for a living host in order to replicate axiomatically sets limits to the amount of damage a virus can do to a host at either individual or population level. Virulence is the quantitative expression of such damage^1–3^: essentially the compromise between the host’s ability to control the virus or its damage versus the ability of the virus to circumvent this control^1^. It is a highly plastic and adaptive trait, particularly through the virus, subject to selection at multiple levels of host organization (molecular, cell, tissue, individual, population, species) with different possible outcomes at the different organizational levels, including levels where the interests of virus and host may be aligned, for example as a weapon in ecological competition with more susceptible hosts^4^. Consequently, virulence evolution is equally varied, resulting in all types of host-virus relationships ranging from parasitic host exploitation by the virus through mutualism to enslavement of the virus in function of the host’s interests, including genetic integration of viral and host genomes^5^. The main mechanism for virulence evolution is through the virus genome and in particular its quasispecies, *i.e*. the population of virus genome variants generated by the high rates of mutation and recombination associated with RNA replication^6^. Through the quasispecies, a virus can leverage its small physical genome into a highly variable sequence space (a theoretical representation of all possible variants of a sequence^6^) with which to respond dynamically to adaptive challenges, through a combination of natural selection and complementation^7,8^. Consequently, it is the quasispecies as a whole that is both the target and the result of natural selection^6–9^. The genetic composition of the quasispecies can therefore be visualized as a cloud^7–10^, whose shape shifts depending on which groups of variants are favoured at any one time or place, and whose adaptive potential resides as much in its overall diversity as in the specific capacities of individual variants^8,10,11^.

Of the many factors affecting virulence evolution, the most important is transmission^12,13^. A potent novel transmission route that bypasses the barriers limiting virus distribution and replication at multiple organizational levels, such as vector-mediated transmission, therefore represents a major destabilizing factor affecting the core parameters for virulence evolution, in the conflict between the virus and the host for control of the primary character, and genetic composition, of the virus. Here we investigate aspects of this fundamental conflict for the relationship between honeybees (*Apis mellifera*) and deformed wing virus (DWV), one of the many viruses infecting honeybees and other hymenoptera^14,15^.

The main current biological threat to the western honeybee is a recently acquired exotic parasitic mite, *Varroa destructor*^16^, largely because it vectors lethal epidemics of honeybee viruses^16–18^ that, in the absence of this mite, are relatively innocuous^16^. The principal varroa-transmitted virus is currently DWV^14^. There has long been a lively debate^19^ as to whether the severe pathology caused by DWV is primarily due to genetic adaptation by the virus towards increased virulence^20–22^ or due to excessive virus titres^17,23–25^ precipitated by this potent, novel transmission route^25–28^. Of course, these two alternatives are not mutually exclusive. It has been conclusively shown that DWV is directly responsible for the deformed wing symptoms^25,29,30^; that these symptoms are directly related to both the amount of virus and the transmission route^14,22,24,25^, and that the different major DWV variants (and especially their recombinants) have different replicative capacities in both honeybees and *V. destructor*^20,21,31–33^, as well as different relationships with honeybee pathology at individual bee^24,34^ and colony level^20,21,31,35^. What is less well known is how the virus adapts genetically during these transitions and transmissions.

It is in this context that the following experiments were conducted. In 2008, we discovered several DWV-infected colonies from Leksand, well beyond the *V. destructor* expansion front in Sweden, with a confirmed complete absence of mites and no known prior contact with bees from mite-infested regions^36^. This discovery allowed us to examine a possibly primordial DWV quasispecies (*i.e*. a local DWV quasispecies present prior to the introduction of *V. destructor*) and to test certain hypotheses^19,27,37^ for DWV adaptation during the early establishment of *V. destructor* infestation, specifically whether the DWV quasispecies would adapt genetically to this new and highly potent transmission route during serial transmission and whether these genetic changes had any bearing on DWV pathology, independent of the known effects of DWV titre^25^ and transmission route^25,30,38,39^. The results presented here concern primarily the evolution of the DWV quasispecies during serial transmission. The relationship between DWV quasispecies composition and DWV pathology will be presented separately.

## Results

### Serial DWV-A transmission produces distinct waves of attenuated symptoms

**Figure 1:**
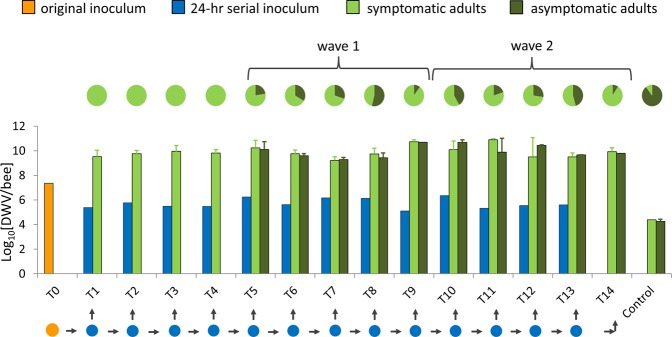
DWV titres and proportions of symptomatic and asymptomatic bees during serial transmission. Changing proportions of symptomatic (light green) and asymptomatic (dark green) adult bees for the entire serial transmission series, with the two waves of increasing and decreasing proportions of asymptomatic adults highlighted, plus corresponding DWV titres in both the serial 24-hour inocula and the adult propagation progeny. The original T0 inoculum is orange. The control bee column describes pooled data of individual mock-injected bees from all the transmission passages. The error bars show the standard error.

The two surviving Leksand colonies^36^ were placed in quarantine in autumn 2009 and moved to Uppsala in summer 2011 for experimentation. A single DWV symptomatic adult from one of these colonies was used as the original source inoculum. Mite-mediated transmission was simulated by microinjection of controlled amounts of virus into pink-eyed honeybee pupae^40^, which were either left to complete development to adults or sacrificed after 24 hours to provide the inoculum for the next transmission (Supplementary Fig. S1). While DWV-B replicates inside varroa^22,25^, DWV-A does not^41^, such that the microinjection accurately mimics the mechanical vectoring of DWV-A by varroa^41^. The serial transfers produced mostly DWV-symptomatic adult bees, confirming the direct causal relationship between DWV and its symptoms^25,29,30,34^, but with occasional ‘waves’ of asymptomatic bees in varying proportions (Fig. 1).

### Symptom attenuation in adult bees is unrelated to DWV-A titre, consensus sequence or quasispecies composition

The symptomatic and asymptomatic bees at each transmission had similar DWV titres, which were at least 5 orders of magnitude larger than for the mock-inoculated control bees (Fig. 1) while sequence analysis showed that the DWV in the mock-inoculated samples was identical to the DWV of the serial inocula (Supplementary Fig. S2), and thus most likely derived from experimental contamination, rather than from pre-existing local DWV in the pupae. These observations indicate that the elevated DWV titres at each transmission were due to the virus present in the serial inocula. The full DWV genome sequences of the first (T0) and final (T13) 24-hour transmission inocula were 100% identical, indicating that there had been no long-term genetic adaptation by DWV to simulated serial varroa transmission. These DWV sequences were also quite distinct from the original Leksand DWV sequences (Supplementary Fig. S2), casting a degree of uncertainty over the provenance of the original T0 inoculum. The absence of reliable biogeographic clustering throughout the tree (and in DWV phylogenies in general^28,42,43^) makes it furthermore impossible to either confirm or deny a possible Leksand origin for the T0 inoculum, and thus its primordial status. The question of whether a naïve, primordial DWV quasispecies would adapt genetically to varroa-mediated transmission therefore remains unanswered.

### The adult DWV-A quasispecies composition is shaped by strong, conservative selection from initially diverse post-inoculation quasispecies

**Figure 2:**
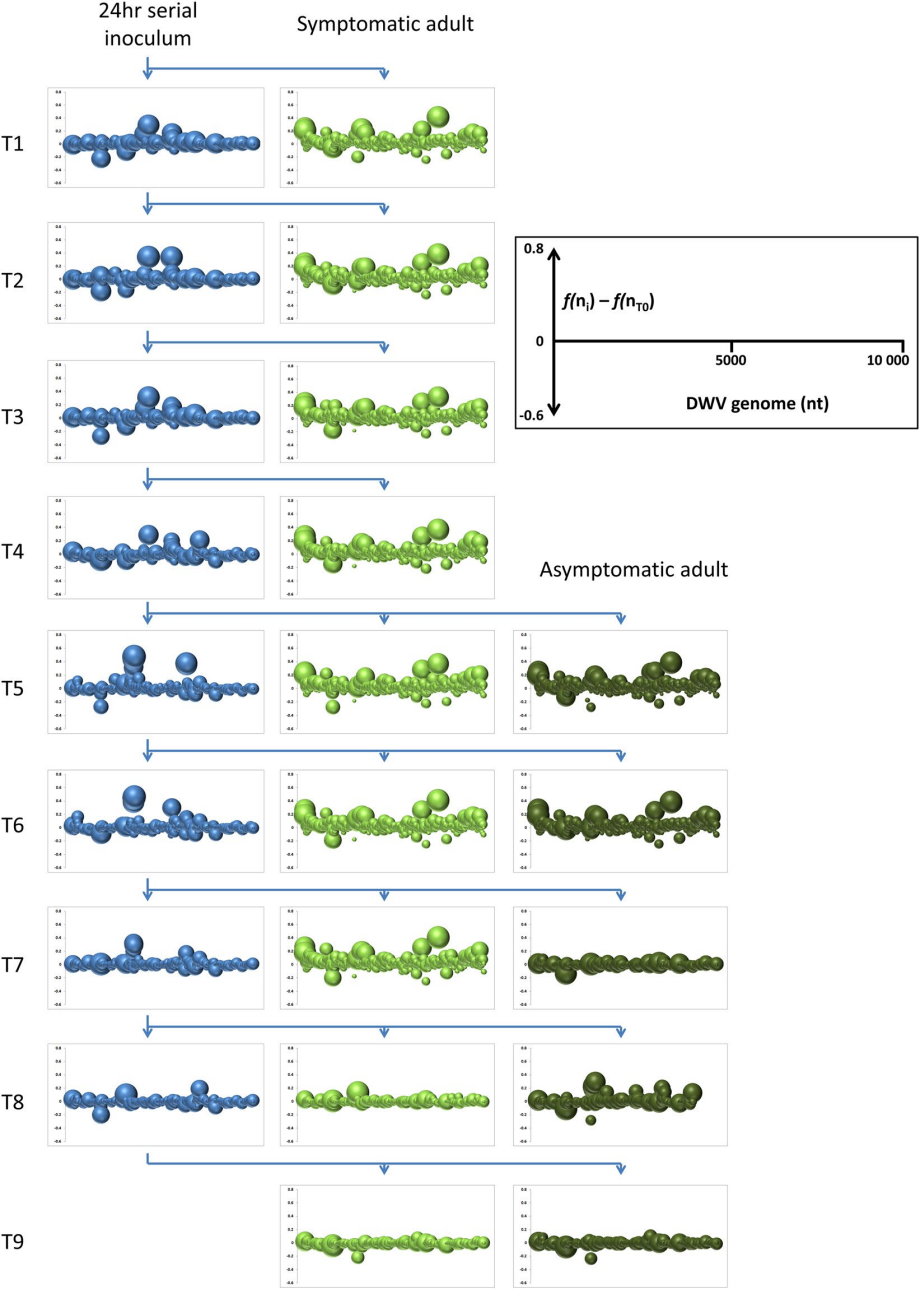
Graphical representation of DWV quasispecies composition change during transmission. Bubble-graph representation of the difference in frequency (*f*) of the consensus nucleotide (*n*) at each nucleotide position along the 10 kb DWV genome (nt) between the experimental sample (*f(n*_*i*_)) and the original T0 inoculum (*f(n*_*T0*_)), shown for each of the successive 24-hour transmission inocula (blue; T1–T8) and their corresponding 7-day symptomatic (light green) and asymptomatic (dark green) adult transmission progeny. The size of each bubble represents the extent of the change in the sub-consensus nucleotide frequency along the DWV genome, *i.e*. those quasispecies with the smallest bubbles and the least amplitude are most similar to the original T0 inoculum.

In the absence of consensus DWV sequence change, we decided to investigate possible changes in the genetic composition of the DWV quasispecies through the first wave of asymptomatic bees, in both the 24-hour transmission inocula and the resulting (a)symptomatic adult bees. The DWV quasispecies in each sample was represented by a matrix of Single Nucleotide Polymorphism (SNP) frequencies along the entire DWV genome, which allows all the major types of variation found in viral quasispecies (point mutations, deletions, major strains and recombinants^6–8^) to be represented. However, the DWV quasispecies analysed in these experiments was grouped around only one of the major DWV strains (DWV-A^44^), and therefore involves a narrower range of genetic variability and more refined resolution of effects than quasispecies consisting of multiple major strains and their recombinants^20–22,31,32,35^. Although the DWV consensus sequence remained constant and near-identical to the T0/T13 consensus sequence throughout this first wave of transmissions and adult progeny (Supplementary Fig. 2), radical and consistent patterns of change were observed in the DWV quasispecies underlying these consensus sequences, as represented by changes in the frequency distributions of the four nucleotides (or a deletion) along the DWV genome, relative to the original inoculum (Fig. 2). The principal distinction is between the relatively diverse range of quasispecies for the 24-hour serial pupal inocula (blue) and the more uniform range for the (a)symptomatic adult progeny of each serial transfer (green). Particularly striking is the strong uniformity of the adult bee quasispecies, both between symptomatic (light green) and asymptomatic (dark green) bees of each serial transfer and, more interestingly, also between different serial transfers, despite a diverse range of 24-hour quasispecies (blue) serving as the inocula for these serial transfers. The overall nucleotide diversity was similar for the DWV quasispecies of the serial inocula and the adult bees, but distributed differently within the genome, with consistent evidence from multiple indicators (Table 1) that the adult quasispecies are shaped mostly by negative selection, while there is little evidence of any selection (positive, negative or neutral) within inocula quasispecies. This observation does not preclude the possibility of positive, directional selection during quasispecies development; rather that the evidence for this can be quickly obscured by subsequent quasispecies changes.

**Table 1:** DWV quasispecies diversity and selection indices according to host developmental status.

Sample Type	Diversity	Selection Indices					
	Nucleotide diversity (π)	Tajima’s D	Fu and Li’s D	Fu and Li’s F	Codon-Based Z Test of Selection (Nei-Gojobori)		
					Neutral	Positive	Negative
Pupae (N = 8)	0.011	−0.110	−0.240	−0.234	*P* = 0.111^ns^	*P* = 1.00^ns^	*P* = 0.058^ns^
Adult bees (N = 13)	0.007	−0.815	−1.190	−1.247	***P*** = 0.013^*^	*P* = 1.00^ns^	***P*** = 0.006^*^

Estimates of the nucleotide diversity and selection indices for the DWV quasispecies from the 24-hour serial inocula (pupae) and the 7-day (a)symptomatic adult transmission progeny (adult bees). Negative values of Tajima’s D^87^ and Fu and Li’s D and F^88^ selection indices signify an excess of low frequency polymorphisms, indicating expansion of the population size as well as negative (purifying) selection. P-values in the Codon-Based Z Test of Selection refer to the probability of rejecting the null hypothesis of strict-neutrality (equal indices of synonymous and non-synonymous substitutions) in favour of the alternative hypothesis (positive or negative selection). Values of P > 0.05 were considered non-significant (ns). The values were estimated from the sub-consensus sequences of the different quasispecies. Only characters with a Minor Allele Frequency (MAF) ≥ 0.02 were included in the sub-consensus sequences.

### DWV-A quasispecies composition is linked to developmental state rather than inoculation history

**Figure 3:**
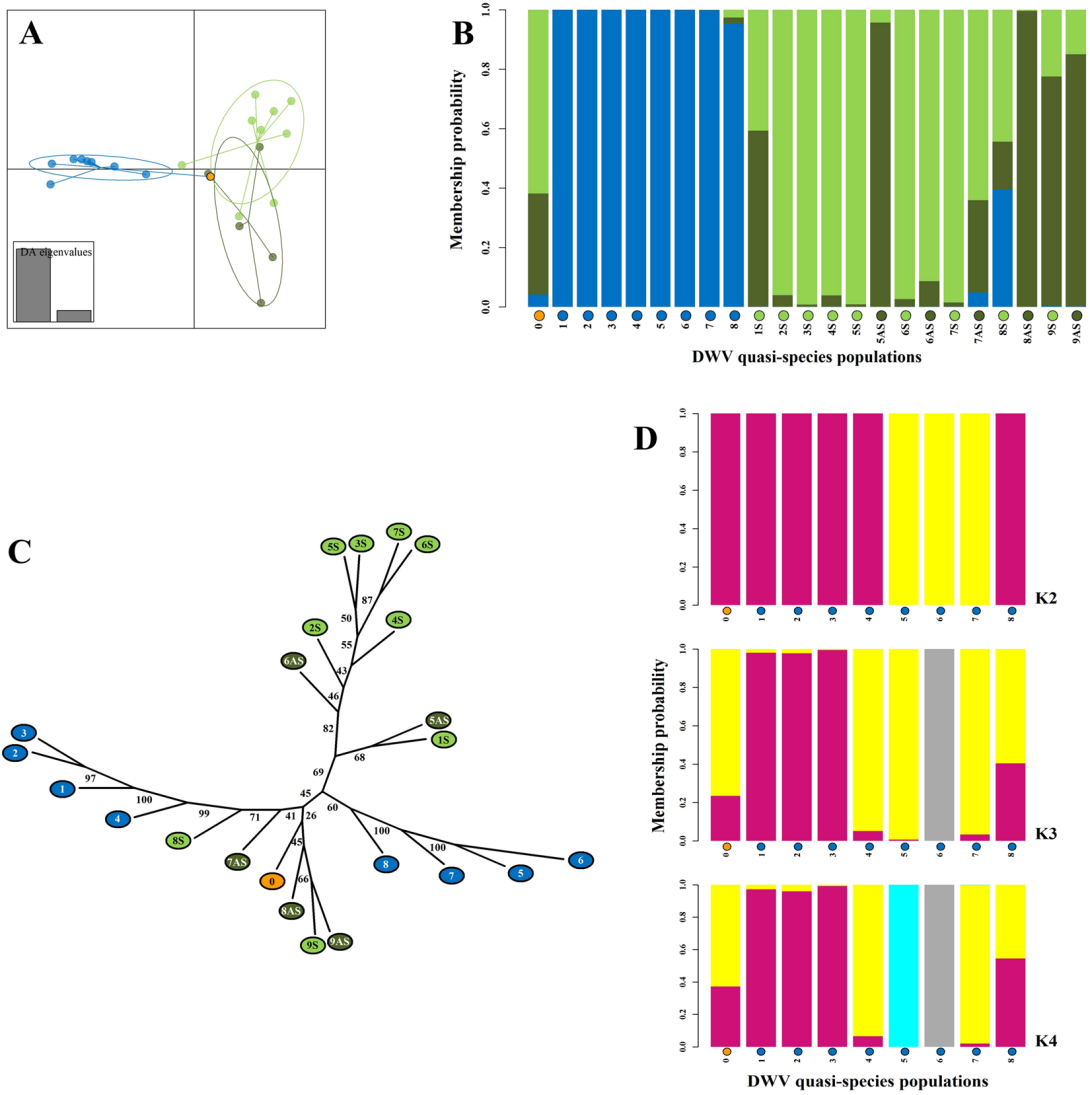
Clustering analyses of the DWV quasispecies. Discriminant Analysis of Principal Components (DAPC) and genetic clustering analyses of the DWV quasispecies nucleotide frequency data. Alleles were considered polymorphic with a Minor Allele Frequency (MAF) ≥ 0.02, resulting in 1,212 characters (about 12% of the DWV genome) from the 23 DWV quasispecies analysed. (**A)** Bounded scatterplot locating the 23 different quasispecies in a two-dimensional space, based on the principal data elements distinguishing the three sample types: inocula (blue), symptomatic adults (light green), asymptomatic adults (dark green). The orange dot marks the original T0 inoculum. (**B)** Compoplot showing the proportional genetic assignments of each quasispecies to the three genetic clusters defined by sample type. (**C)** Phylogenetic tree inferred from the allele frequency data using the Neighbour Joining algorithm implemented in PHYLYP (v.3.65c)^93^. Branch lengths represent genetic distance. Numbers along the branches represent statistical support for the branch, as determined by bootstrapping analysis (1,000 replicates). The sample types and order are marked by colour and number to identify each sequential 24-hour serial transmission inoculum (1–8: blue), and the matching symptomatic (-S: light green) and asymptomatic (-AS: dark green) adult inoculation progeny. (**D)** Unconstrained genetic clustering patterns with increasing K-clusters among DWV quasispecies of the 24-hour transmission inocula. The serial transmission proceeds from left to right, with the original T0 inoculum marked orange.

The most parsimonious explanation is that injection caused a rapid burst of replication that generated extensive variation, seen in the 24-hour inocula, followed by largely negative selection as replication slowed down, resulting in a convergence of quasispecies shapes^45^, as seen in the adult bees. The surprising feature is the strength and consistency of this convergence through multiple serial transmissions, considering the diversity of shapes and variation generated by the 24-hour inocula. These observations are confirmed by more formal analyses (Fig. 3). Despite the much longer evolutionary distance between different adult bees than between any bee and its 24-hour inoculum, the adult bee quasispecies cluster together, with little difference between symptomatic and asymptomatic bees, and well separated from the cluster of 24-hour inocula (Fig. 3A,B). Of special interest here is that the original T0 inoculum, classified in the DAPC as an inoculum but derived from an adult bee, locates both in the plot (Fig. 3A) and by genetic assignment (Fig. 3B) very clearly with the adult quasispecies rather than with its pre-assigned group, the inocula. This is the first indication that the host’s status may be highly influential in determining the DWV quasispecies composition^46^. A phylogenetic analysis of the composition of the different quasispecies shows that there is no correspondence between the inferred phylogenetic relationships, based on shared genetic signatures, and their known parentage through serial transmission, particularly for the adult bee quasispecies (Fig. 3C). This attests to the extreme plasticity of the quasispecies and its capacity for rapid and extensive change in composition, thereby obscuring the stable phylogenetic signal required for successful reconstruction of the history of descent. Even though the potential for change exists at every transmission event, shown by the extensive initial changes to the DWV quasispecies 24 hours after injection, the end result in the adult host is a conserved standard shape. A similar conservation of quasispecies shape is also observed through the multiple serial transmissions, mimicking sequential transmission within a colony, since the adult DWV quasispecies after the seventh serial transmission, particularly 7AS, 8 S, 8AS, 9 S and 9AS, were very similar to the original T0 inoculum (Figs. 2, 3C). Both the adult quasispecies and those of the 24-hour inocula show a slight phylogenetic clustering by their serial transmission number, with robust assignment of the individual taxa (Fig. 3C), suggesting that at least some of the genetic signatures in the inocula were transmitted through several consecutive transfers to subsequent inocula, while different genetic signatures were retained through successive transfers in the adult quasispecies that develop from these inocula (Fig. 3D, Supplementary Fig. S3). However, the fleeting volatility of such signatures, both between transfers and during the transformation of the quasispecies towards the shapes encountered in adult bees, makes it difficult to capture them by clustering analyses, unless they are persistent and extensive.

## Discussion

Despite many decades of co-existence between honeybee, *V. destructor* and DWV, in millions of colonies and billions of honeybees world-wide, the pathology and virulence of DWV remain stubbornly linked to direct vectored transmission by *V. destructor* in developing pupae^14,22–25,34^, with DWV pathology, virulence and titres all disappearing from honeybee colonies when varroa is removed^23,29^. This suggests that DWV genetic adaptation to transmission by *V. destructor* is either non-existent, and DWV virulence largely determined by the transmission route and viral titres^25^, or ephemeral; present only during varroa-mediated transmission with the DWV quasispecies rapidly returning to a default genetic shape and composition when varroa transmission is removed. The evidence accumulating thus far strongly favours the second mechanism, *i.e*. the ability of the DWV quasispecies to adapt very rapidly to different selective challenges through compositional changes to the genetic structure of its quasispecies. Most of this evidence comes from studies on the relative virulence of DWV-A and DWV-B, the two major strains of DWV^14,44^. Both strains are capable of producing symptoms and disease upon injection into pupae, either by varroa or experimental inoculation^25,34,47^, but DWV-B is considerably more virulent than DWV-A at both individual bee^22^ and colony level^21^ and is more closely associated with varroa^22,48^ and winter mortality^21,49^. Recombinants between the two strains are possibly even more virulent than either parent^20,31,32^^,^ and have made it possible to map this virulence to the RNA-dependent RNA polymerase region of DWV-B^22^. However, virulence also comes at a cost, both individual^22,50–53^ and social^54–56^, which may explain why the natural incidence of DWV-A/DWV-B recombinant viruses is low^57^, despite having many selective advantages over their parents in laboratory studies^20,22^, and why natural recombinants often contain the “avirulent” DWV-A polymerase region^20,31,32,47,58,59^. For example, both DWV-A and DWV-B are transmitted by varroa, but only DWV-B also replicates in varroa brain tissues^41,48,60^. While this enhances transmission^20^, it also impairs varroa neurological function^61^, which is essential for host finding and reproduction, thus neutralizing some of DWV-B’s selective advantage over DWV-A. A similar, but inverse, cost-benefit relation exists in honeybees. DWV-B is more virulent than DWV-A in honeybee pupae^22^, and thus more likely to be detected and removed by honeybee hygienic behaviour^50,52^ and, at a higher level, by elevated colony winter mortality^21,49^. However, DWV-B also impairs to a greater extent than DWV-A the cognitive behaviour of adult bees^22^, an essential prerequisite for effective hygienic behaviour^51^, increasing the chance of DWV-infected pupae escaping hygienic surveillance^50^. In both varroa and honeybees therefore, the cost of DWV-B’s higher virulence is greater neurological impairment of its host, which can be either beneficial or detrimental to the virus depending on host, developmental stage and colony context. This is a natural foundation for the appearance of multiple strains adapted to different contexts. Since there is little difference between DWV-A and DWV-B in pathogenicity^47,49^, titres^20,33,47,49^ or adult mortality^22^, the relative persistence of DWV-A, DWV-B and/or their recombinants in bee colonies may well be driven largely by pupal mortality and the effectiveness of social hygiene^50^, where the strains differ most.

Like others before us, we also could not distinguish DWV quasispecies from symptomatic and asymptomatic adult bees^24^ and found the DWV quasispecies dynamics to be dominated by rapid selective processes^20,22,33^. Results elsewhere suggest a limited role for the cooperative structure of viral quasispecies^8,10,62^ in DWV quasispecies dynamics^33,60^. No long-term genetic adaptation to transmission by injection was observed in our experiments, suggesting that our DWV-A quasispecies was suitably adapted to mechanical vectoring^41^.

We identified the host developmental state as an additional, distinct and powerful modulator of DWV quasispecies composition, after serially transmitting DWV-A into successive generations of white-eyed pupae, and following the fate of the DWV-A quasispecies through subsequent development into symptomatic or asymptomatic adult bees. The waves of asymptomatic adult bees in the serial transmission progeny suggest that the appearance of these attenuated symptoms has a systemic origin. This could have been host or virus-derived factors interfering with symptom development^63,64^ that were transmitted through several serial inocula until dissipated. Alternatively, the clustering may reflect systematic variation in the molecular condition of the pupae used for experimentation. These were also harvested serially and could have been conditioned by their immediate environment, such as nutrition^65,66^ or the presence of other bee viruses^47,67,68^, which can affect susceptibility to DWV infection^64,66^, its pathology^47^, tolerance of its effects^65^ or interfere with DWV replication^26,67^ or transmission^68^. These alternatives will be investigated further in future experiments and more detailed analyses of the samples.

The consistent clustering of the DWV quasispecies genetic profiles by the bee developmental state, rather than by serial inoculation history or sample hierarchy, suggest that the shape of this DWV-A quasispecies is strongly influenced by the proximal molecular-physiological conditions of its host after transmission, represented here by the difference between the pupal and adult developmental states. Such strong host control over the genetic character of viral quasispecies has been demonstrated repeatedly in other experimental systems, usually through predictable and reversible changes associated with transfer between different hosts^46,69^ or host tissues^70–72^. Despite minor indications that some of the DWV-A quasispecies genetic signatures are transferred serially between inocula, there is no concrete evidence of any progressive directional change in the 24-hour inocula quasispecies composition that would indicate an adaptation to transmission by injection, either from the indicators of selection (Table 1) or from the genetic composition of the quasispecies (Fig. 3D). Both theoretical^9,62^ and experimental studies^22,45,46,73,74^ have shown that once produced, favourable genetic features or shapes can become fixed very rapidly in a viral quasispecies, often within a few serial passages. Such rapid genetic adaptation has also been inferred from analysing the fate of different DWV master variants and their recombinants^20,21,34^, with different replication characteristics^21,33^, in different transmission environments^20–22,31^ or hosts^22,75,76^. The current experiments were therefore of sufficient duration and scope to have identified genetic adaptations specific to the transmission route, if these were either available in the original DWV-A quasispecies or produced during the experiment.

These experiments also provide a possible mechanistic explanation for those instances where adaptation to transmission route^17^ or host species^75^ did not result in divergence of the DWV consensus sequence if, as was the case here, the adaptability of the virus involved primarily the re-distribution of its internal genetic variability, rather than fixed changes to the consensus sequence or competition between major strains and recombinants^20–22,33^. The results also imply that any features of the DWV quasispecies present during early wing development that may be relevant for the DWV-symptomatic changes in wing morphology will have largely disappeared from the quasispecies by the time the symptoms become visible in the adult bees, helping explain why no genetic markers for DWV symptoms have yet been identified on the DWV genome^24,34^. Higher resolution mapping of the transformation of the DWV quasispecies during pupal development, tissue tropism or host-range studies would provide greater clarity of the extent of host control over the DWV quasispecies, while the mechanisms of such host-specific control would require more comprehensive analyses of the RNA samples produced from such studies. The rapid adaptability and resilience of the DWV quasispecies also helps explain why DWV functionally reverts to its low-titre, benign status when mite-mediated transmission is removed^23^, if this represents the preferred, ancestral relationship between virus and host. Such consistent reversal to low virulence, or to a common quasispecies shape (as shown here), hints at a more complex host-pathogen relationship than mere pathological exploitation and perhaps other, more mutualistic^77^ roles for DWV to bee life at some level of organization (cell^51^, organ, bee, colony, population) or context (health^14,16,17^, sociality^50^, ecology^4,78^), as one of the predicted possible outcomes of host-pathogen co-evolution^8,27,79^. Since these viruses are part of a shared pathosphere involving other bees, wasps and bumblebees^4,57,78^, the question also has relevance outside the beekeeping community^4,75,78^.

## Methods

### Source of bees and virus

The two surviving colonies in the Leksand study^36^ were transferred in September 2009 from Leksand to an isolated forest area near Forsmark (60.393735, 18.208015), at that time also still beyond the varroa front. When *Varroa destructor* was detected in Forsmark in 2009, the colonies were relocated in March 2010 to a quarantine apiary in Grötlingbo Nature reserve on Gotland (57.121910, 18.419933), 4.8 km from the nearest registered bee colonies, since they could no longer be moved back into varroa-free areas on mainland Sweden or its archipelagos. One year later, in June 2011, the colonies were brought back to Uppsala for experimentation. The DWV starting inoculum (T0), prepared as described below, was obtained from a single symptomatic adult honeybee from one of these colonies. By the end of 2011 both colonies had acquired varroa. A *Varroa destructor*-free and DWV-free honeybee colony from Utö (58.915134, 18.211738), an island in the Swedish archipelago that has never been infested with *V. destructor*, was used to obtain the experimental worker pupae for serial transmission. Throughout the experiments, control worker pupae from this colony tested negative for DWV by RT-qPCR.

### Serial DWV transmission

The serial transmission experiments were conducted during the summer of 2011, according to the experimental design shown in Supplementary Fig. S1. Each serial inoculum was prepared by macerating the worker pupa in 500 µL PBS buffer, homogenizing with 100 µL chloroform and strong vortexing, and clarifying the extract by centrifugation at 13000 rpm for 10 minutes, retaining the supernatant as inoculum and for RNA extraction^40^. At each transmission passage, around 15 pink-eyed, white-bodied pupae were each microinjected laterally in the abdomen, in between the 2^nd^ and 3^rd^ integuments, with 2 µL DWV inoculum obtained from the previous passage, using a 50 µl microsyringe (Hamilton Microliter™ Syringes, Nevada, USA) and very thin, 30 gauge disposable needles. The needles were replaced between individual pupae. The pupae were incubated on Whatman filter paper in disposable petri dishes in an incubator at 35 °C and 80% relative humidity^40^. After a 24 hour incubation, a single random pupa was selected as the DWV inoculum for the next serial passage, while the remaining pupae were incubated a further 6 days to complete their development into adult bees. All collected bees were stored at −80 °C until further use. This procedure was repeated for 14 serial transmissions per trial, and for two independent trials. For technical controls, ten pink-eyed pupae were microinjected with just 2 μL PBS at each transmission. From the serial transmission assays detailed above, all the serial 24-hour inocula pupae and at least one of the corresponding adult transmission progeny were selected for DWV quasispecies analysis through IonTorrent sequencing.

### RNA extraction and reverse transcription

The 24-hour pupal samples were homogenized at room temperature in 500 µL PBS buffer (Phosphate Buffered Saline; pH 7.4), of which 100 µL was used for RNA extraction and the remainder stored at −80 °C until further use. The 7-day adult bee samples were homogenized in 200 µL TN buffer (0.1 M Tris pH 7.9, 0.1 M NaCl), of which 50 µL was used for RNA extraction and the remainder stored at −80 °C. RNA was extracted from these 24-hour and 7-day sample homogenates using NucleoSpin® RNA Kit (Macherey-Nagel, Düren, Germany) and was eluted into 50 μL nuclease-free water. The approximate RNA concentration was determined by NanoDrop. Reverse transcription was performed in 20 µL reactions containing approximately 2.5 µg RNA, 10 mM dNTPs, 100 µM random hexamer primers and 400 units SuperScript™ III RT (Invitrogen™, Life Technologies Corporation, New York, USA). The resulting cDNA was purified using the High Pure PCR purification Kit (Roche Life Sciences, Penzberg, Germany), eluted into 100 μL elution buffer (10 mM Tris-HCl, pH 8.5) and stored at −80 °C until further use.

### RT-qPCR quantification of honeybee viruses

The levels of several common honeybee viruses (Acute bee paralysis virus – ABPV; Israeli acute paralysis virus – IAPV; Kashmir bee virus – KBV; Black queen cell virus – BQCV; Chronic bee paralysis virus – CBPV; Deformed wing virus strain A (DWV-A) and strain B (DWV-B); Sacbrood virus – SBV; Slow bee paralysis virus – SBPV; Bee Macula-like virus - BeeMLV) were quantified by qPCR of the cDNA using previously described protocols and primers^23^, as was the *Apis mellifera* β-actin mRNA as an internal reference gene for quality control and data Normalization^40,80^ (Supplementary Table S1). The reactions were run in triplicate, using KAPA SYBR® FAST Universal qPCR kit (KAPA Biosystems), 0.3 μL cDNA and 0.2 μM each of forward and reverse primer in a 12 μL reaction volumes. The qPCR was processed in an ECO™ Real-Time PCR machine (Illumina, SD, USA) with the following qPCR cycling profile: 3 min at 95 °C followed by 40 cycles of [3 sec at 95 °C - 30 sec at 57 °C - data collection]. The amplification was followed by a melting curve analysis by reading the fluorescence at 0.5 °C increments from 55 °C to 95 °C, to verify the specificity of the qPCR products. Apart from DWV, only low levels of BQCV and SBV were detected in these samples, which is consistent with current knowledge of the viruses found naturally in Swedish honeybees^23,81^.

### DWV whole genome amplification

The whole DWV genome was amplified from one symptomatic adult bee and (where possible) one asymptomatic adult bee from each of the first 9 serial transmissions, as well from the corresponding 24-hour serial transmission inocula, each of which was also derived from a single bee. The DWV genome was amplified in four overlapping sections covered by primer-pairs F27/B1806, F1725/B3095, F3018/B7295 and F7243/B10101 (Supplementary Table S1). In a few instances fragment F3018/B7295 was replaced by three smaller fragments covered by primer-pairs F3018/B4329, F4220/B5625 and F5625/B7295; and fragment F7243/B10101 by smaller fragments F7243/B8794 and F78688/B10101 (Supplementary Table S1). These primer pairs were designed conservatively to amplify all known DWV variants^44^. PCR was performed using the high fidelity KAPA HiFi™ HotStart kit (Kapa Biosystems Ltd., London, UK), to minimize the introduction of PCR-derived errors. The reactions contained approximately 0.2 µg cDNA, 10 mM dNTPs, 0.3 µM of each primer and 0.5 units of KAPA HiFi™ HotStart DNA polymerase in 25 µL volumes. The reaction was processed in a ECO™ Real-Time PCR machine (Illumina, SD, USA) with the following qPCR cycling profile: 2 min at 95 °C, followed by 35 cycles of [20 sec at 98 °C - 20 sec at 56 °C – 3 min at 72 °C], followed by 2 min at 72 °C. The fragments were purified using the High Pure PCR purification Kit (Roche Life Sciences, Penzberg, Germany), and the fragment sizes were confirmed by electrophoresis through a 1.2% agarose gel, stained in freshly prepared GelRed^TM^ for 30 min and visualized under UV light. Although the diagnostic DWV assays detected very low amounts of DWV in some of adult bees developing from mock/PBS-inoculated pupae, the DWV whole-genome amplification assays failed for the Control samples, except for the 694 bp Lp fragment.

### Sequencing

First, the DWV whole-genome PCR fragments of the T0 and T13 24-hour inocula (Fig. 1; Supplementary Fig. S1) were sequenced using Sanger technology and primer-walking, to identify any consensus changes over the entire adaptation experiment. The 694 bp DWV-Lp gene fragment of the Control sample was also sequenced with Sanger technology. When the T0 and T13 DWV full genome sequences were found to be 100% identical by Sanger sequencing, a NGS strategy was designed to assess the variability underlying this consensus sequence uniformity. NGS sequencing was performed using the Ion PGM system (ThermoFisher) at the National Genomics Infrastructure in Uppsala. 0.1–1 µg DNA from each sample was fragmented using the S2 system (Covaris). The fragment ends were repaired and ligated to unique bar-coded adaptors using the Applied Biosystems Library builder (ThermoFisher). The adaptor-linked fragments were amplified using the Ion Xpress™ Plus gDNA Fragment Library Preparation protocol and selected for a 470 bp target size range (Blue Pippin^TM^, Sage Science). Library size and concentration were assessed by a Bioanalyzer High Sensitivity Chip (Agilent Technologies) and the Fragment Analyzer system (Advanced Analytical). The DNA samples were pooled together in sets of nine, followed by template preparation using the Ion PGM™ Template OT2 400 Kit on the Ion OneTouch™ 2 system (ThermoFisher). Samples were then sequenced on the Ion PGM™ System with Ion PGM™ Sequencing 400 Kit on Ion 316v2 chips (ThermoFisher) aimed at a 400 bp read length.

### Data analysis

The IonTorrent sequence reads were first filtered by bar-code, to identify the sample origin, and then mapped to reference sequences AY292384 (DWV-A), AY251269 (DWV-B) and CEND01000001 (DWV-C) using Tmap included in TorrentSuite 3.6.2 with LifeTechnologies recommended parameters. Variability data and consensus sequences were created using mpileup from SAMtools (v.0.1.8)^82^ and an in house script. This resulted in a numerical file for each sample containing the frequencies of each nucleotide or a deletion at each position of the DWV genome between nucleotides 10 and 10,120 of the mapping reference sequence for all samples, as well as the sequencing coverage across the DWV genome (average/max/min per sample = 8952×/14292×/2772×), thus capturing the full genetic complexity of the DWV quasispecies^83^. These files form the basis for the data analyses. Statistical analyses were conducted in R unless stated otherwise.

### Diversity and selection analyses

The DWV consensus sequences of the 23 NGS sequenced samples were, with the exception of 3 individual SNPs, identical among each other (Supplementary Fig. S2). Their combined consensus sequence was also identical to the T0 and T13 Sanger sequences, and this combined consensus sequence has been submitted to GenBank under accession number MN746311. The genetic analyses therefore concern primarily the distribution of the genetic variation underlying these consensus sequences, and how they are related to each other. A sub-consensus sequence database was created for each individual sample by calling the second most frequent nucleotide at all polymorphic nucleotide sites after filtering out indels (insertions/deletions) and those nucleotides with frequencies lower than 0.2% or higher than 50%. The consensus and sub-consensus sequences thus obtained were aligned using MEGA^84^ and saved in NEXUS format. Molecular evolutionary analyses were conducted using DnaSP (v5)^85^ and MEGA. Genetic variability from the coding region (polyprotein) within inocula and 7-day sample populations was estimated as nucleotide diversity (π)^86^, consisting of the average number of nucleotide differences per site between all pairs of analyzed sequences, using DnaSP. Similarly, Tajima’s D^87^ and Fu and Li’s D and F^88^ were used to test for neutrality and to infer positive (beneficial) or negative (purifying) selection as implemented by DnaSP.

The Codon-based Z test of Selection was performed calculating the number of synonymous substitutions per synonymous site (d_S_) and the number of non-synonymous substitutions per non-synonymous site (d_N_) between inocula and 7-day sample populations using the Nei and Gojobori’s method^89^ with MEGA in order to reject the null hypothesis of strict neutrality (H_o_: d_S_ = d_N_) and infer, as well, the balance between neutral mutations, negative selection and positive mutations acting on the DWV quasispecies.

### Clustering analyses

The clustering analysis of the genetic variation between the sequenced samples was performed using Discriminant Analysis of Principal Components (DAPC), a model-free method implemented in the adegenet package for R^90,91^. This method relies on reducing a large SNP dataset to a number of uncorrelated variables by Principal Component Analysis (PCA), which are subsequently used by discriminant analysis (DA) to assign the samples to the different genetic clusters by maximizing between-group variance and minimizing within-group variance. The input for the DAPC consisted of frequencies of polymorphic SNPs (columns) for each of the 23 virus quasispecies populations (rows). The data for sample 8AS was based on a reduced genome, since the sequence coverage for the last 1,302 nucleotides at the 3′ end of the genome was below the ≥100x cut-off value for nucleotide frequency calculations (see Fig. 2). The criteria for SNP inclusion was a MAF > 0.02, which is well above the technical error rates for IonTorrent sequencing^92^. Two SNP datasets were constructed. The first dataset included the 23 DWV quasispecies populations, for which there was a total of 1,212 polymorphic SNPs (Fig. 3B). The second dataset included the eight 24-hour transmission inocula, for which there was a total of 773 polymorphic SNPs (Fig. 3D). The sequential K-means clustering algorithm was run in the second dataset for K = 1 to 8 using 1 × 10^6^ iterations per run. The different clustering solutions were compared using the Bayesian Information Criterion (BIC) to identify the optimal number of genetic clusters that describe the data. The optimal K is associated with the lowest BIC value^91^.

### Phylogenetic analysis

The phylogenetic analyses of the SNP frequency data were conducted with various programmes implemented by PHYLYP (v.3.65c)^93^. Nei’s genetic distance^86^ between the 23 DWV quasispecies samples was calculated from the SNP frequencies of the 1,212 characters in the DPC data matrix using the programme ‘Gendist’. This distance matrix was used to construct a phylogenetic topology using the Neighbour-Joining algorithm in the programme ‘Neighbour’ and the consensus tree was built using the programme ‘Consense’. Statistical support for the branches in the consensus topology was provided by 1,000 replicate bootstraps of the data, using the programme ‘Seqboot’. The phylogenetic analysis describing the relationship between the DWV samples from the current experiment and other biogeographic isolates from Sweden and Gotland^14,36,93^ relative to a selection of DWV-A^44,94^ isolates representing Europe and the Middle East^14,20,32,43,58,94,95^, North and South America^33,94,96^ and East Asia^14,97–100^ was inferred using the Maximum Likelihood method and the Tamura-Nei model of evolution^101^, as implemented by MEGA-X^85^, using the DWV-B^44,48^ and DWV-C^44^ reference strains as outgroup sequences. The statistical confidence of each of the nodes was determined by bootstrap analysis involving 500 replicates. Initial tree(s) for the heuristic search were obtained automatically by applying Neighbor-Join and BioNJ algorithms to a matrix of pairwise distances estimated using the Maximum Composite Likelihood (MCL) approach, and then selecting the topology with superior log likelihood value. All positions containing gaps and missing data were eliminated resulting in a total of 694 positions in the final dataset.

## Supplementary information

Supplementary Figures Tables.

## Data Availability

The datasets generated and/or analysed during the current study are available from the corresponding author on reasonable request.

## References

[1] Casadevall A, Pirofski L-A (1999). Host-pathogen interactions: redefining the basic concepts of virulence and pathogenicity. Infect. Immun..

[2] Schmid-Hempel, P. Evolutionary parasitology. O.U. Press, ed. (Oxford University Press, 2011).

[3] Kurath, G. & Wargo, A. R. Evolution of viral virulence: empirical studies. In Weaver, S. C., Denison, M., Roossinck, M. & Vignuzzi, M. (eds.) Virus Evolution: Current research and future directions. 155–214 (Caister Academic Press, 2016).

[4] Koch H, Brown MJF, Stevenson PC (2017). The role of disease in bee foraging ecology. Curr. Opin. Insect Sci..

[5] Holmes EC (2011). The evolution of endogenous viral elements. Cell Host & Microbe.

[6] Domingo E (1998). Quasispecies structure and persistence of RNA viruses. Emerg. Infect. Dis..

[7] Domingo E, Sheldon J, Perales C (2012). Viral quasispecies evolution. Microbiol. Mol. Biol. Rev..

[8] Dolan PT, Whitfield ZJ, Andino R (2018). Mechanisms and concepts in RNA virus population dynamics and evolution. Annu. Rev. Virol..

[9] Elena SF, Sanjuán R (2007). Virus evolution: Insights from an experimental approach. Annu. Rev. Ecol. Evol. Syst..

[10] Lauring AS, Andino R (2010). Quasispecies theory and the behavior of RNA viruses. Plos Pathog..

[11] Vignuzzi M, Stone JK, Arnold JJ, Cameron CE, Andino R (2006). Quasispecies diversity determines pathogenesis through cooperative interactions in a viral population. Nature.

[12] Lipsitch M, Siller S, Nowak MA (1996). The evolution of virulence in pathogens with vertical and horizontal transmission. Evolution.

[13] Clayton DH, Tompkins DM (1994). Ectoparasite virulence is linked to mode of transmission. Proc. R. Soc. Lond. B. Biol. Sci..

[14] de Miranda JR, Genersch E (2010). Deformed wing virus. J. Invertebr. Pathol..

[15] Singh R (2010). RNA Viruses in hymenopteran pollinators: Evidence of inter-taxa virus transmission via pollen and potential impact on non-Apis hymenopteran species. Plos one.

[16] Genersch E (2010). Honey bee pathology: current threats to honey bees and beekeeping. Appl. Microbiol. Biotechnol..

[17] Martin SJ (2012). Global honey bee viral landscape altered by a parasitic mite. Science.

[18] Mondet F, de Miranda JR, Kretzschmar A, Le Conte Y, Mercer AR (2014). On the front line: Quantitative virus dynamics in honeybee (*Apis mellifera* L.) colonies along a new expansion front of the parasite *Varroa destructor*. PLoS-Path..

[19] Neumann P, Yañez O, Fries I, de Miranda JR (2012). Varroa invasion and virus adaptation. Trends Parasitol..

[20] Ryabov EV (2014). A virulent strain of deformed wing virus (DWV) of honeybees (*Apis mellifera*) prevails after *Varroa destructor*-mediated, or *in vitro*, transmission. Plos Path..

[21] McMahon DP (2016). Elevated virulence of an emerging viral genotype as a driver of honeybee loss. Proc. R. Soc. Lond. B.

[22] Gisder S, Möckel N, Eisenhardt D, Genersch E (2018). *In vivo* evolution of viral virulence: switching of deformed wing virus between hosts results in virulence changes and sequence shifts. Environ. Microbiol..

[23] Locke B, Forsgren E, Fries I, de Miranda JR (2012). Acaricide treatment affects viral dynamics in *Varroa destructor*-infested honey bee colonies via both host physiology and mite control. Appl. Environ. Microbiol..

[24] Brettell LE (2017). A comparison of deformed wing virus in deformed and asymptomatic honey bees. Insects.

[25] Möckel N, Gisder S, Genersch E (2011). Horizontal transmission of deformed wing virus: pathological consequences in adult bees (*Apis mellifera*) depend on the transmission route. J. Gen. Virol..

[26] Remnant EJ, Mather N, Gillard TL, Yagound B, Beekman M (2019). Direct transmission by injection affects competition among RNA viruses in honeybees. Proc. R. Soc. B.

[27] Cressler CE, McLeod DV, Rozins C, van den Hoogen J, Day T (2016). The adaptive evolution of virulence: a review of theoretical predictions and empirical tests. Parasitology.

[28] Wilfert L (2016). Deformed wing virus is a recent global epidemic in honeybees driven by Varroa mites. Science.

[29] Bowen-Walker PL, Martin SJ, Gunn A (1999). The transmission of deformed wing virus between honeybees (Apis mellifera L.) by the ectoparasitic mite *Varroa jacobsoni* Oud. J. Invertebr. Pathol..

[30] Yue C, Genersch E (2005). RT-PCR analysis of Deformed wing virus in honeybees (*Apis mellifera*) and mites (*Varroa destructor*). J. Gen. Virol..

[31] Moore J (2011). Recombinants between Deformed wing virus and Varroa destructor virus-1 may prevail in Varroa destructor-infested honeybee colonies. J. Gen. Virol..

[32] Dalmon A (2017). Evidence for positive selection and recombination hotspots in Deformed wing virus (DWV). Sci. Rep..

[33] Ryabov EV (2019). Dynamic evolution in the key honey bee pathogen deformed wing virus: Novel insights into virulence and competition using reverse genetics. PLoS Biol..

[34] Tehel A (2019). The two prevalent genotypes of an emerging infectious disease, Deformed wing virus, cause equally low pupal mortality and equally high wing deformities in host honey bees. Viruses.

[35] Mordecai GJ (2016). Superinfection exclusion and the long-term survival of honey bees in Varroa-infested colonies. The ISME Journal.

[36] Forsgren E, Fries I, de Miranda JR (2012). Adult honey bees (*Apis mellifera*) with deformed wings discovered in confirmed *Varroa*-free colonies. J. Apic. Res..

[37] Bolker BM, Nanda A, Shah D (2010). Transient virulence of emerging pathogens. J. Roy. Soc. Interface.

[38] Yue C, Schröder M, Gisder S, Genersch E (2007). Vertical-transmission routes for deformed wing virus of honeybees (Apis mellifera). J. Gen. Virol..

[39] de Miranda JR, Fries I (2008). Venereal and vertical transmission of deformed wing virus in honeybees (Apis mellifera L.). J. Invertebr. Pathol..

[40] de Miranda, J. R. *et al*. Standard methods for virus research in *Apis mellifera*. In: *The COLOSS BEEBOOK, Volume II: standard methods for Apis mellifera pest and pathogen research* (Dietemann, V., Ellis, J. D. & Neumann, P. Eds.) Chapter 13. IBRA, Treforest, UK (2013).

[41] Posada-Florez F (2019). *Deformed wing virus* type A, a major honey bee pathogen, is vectored by the mite *Varroa destructor* in a non-propagative manner. Sci. Rep..

[42] Berényi O (2007). Phylogenetic analysis of Deformed wing virus genotypes from diverse geographic origins indicates recent global distribution of the virus. Appl. Environ. Microbiol..

[43] Haddad NJ (2017). Distribution and variability of deformed wing virus of honeybees (*Apis mellifera* L.) in the Middle East and North Africa. Insect Sci..

[44] Mordecai GJ, Wilfert L, Martin SJ, Jones IM, Schroeder DC (2016). Diversity in a honey bee pathogen: First report of a third master variant of the Deformed wing virus quasispecies. The ISME Journal.

[45] Cuevas JM, Elena SF, Moya A (2002). Molecular basis of adaptive convergence in experimental populations of RNA viruses. Genetics.

[46] Schneider WL, Roossinck MJ (2001). Genetic diversity in RNA virus quasispecies is controlled by host-virus interactions. J. Virol..

[47] Dubois E (2020). Outcomes of honeybee pupae inoculated with deformed wing virus genotypes A and B. Apidologie.

[48] Ongus JR (2004). Complete sequence of a picornalike virus of the genus Iflavirus replicating in the mite *Varroa destructor*. J. Gen. Virol..

[49] Natsopoulou ME (2017). The virulent, emerging genotype B of deformed wing virus is closely linked to overwinter honeybee worker loss. Sci. Rep..

[50] Mondet F (2016). Specific cues associated with honey bee social defence against *Varroa destructor* infested brood. Sci. Rep..

[51] Mondet F (2015). Antennae hold a key to Varroa-sensitive hygiene behaviour in honey bees. Sci. Rep..

[52] Schöning C (2012). Evidence for damage-dependent hygienic behaviour toward *Varroa destructor* parasitised brood in the western honey bee, *Apis mellifera*. J. Exp. Biol..

[53] Iqbal J, Mueller U (2007). Virus infection causes specific learning deficits in honeybee foragers. Proc. Roy. Soc. B: Biol. Sci..

[54] Sumpter DJT, Martin SJ (2004). The dynamics of virus epidemics in Varroa-infested honey bee colonies. J. Anim. Ecol..

[55] Benaets K (2017). Covert deformed wing virus infections have longterm deleterious effects on honeybee foraging and survival. Proc. Roy. Soc. B..

[56] Wells T (2016). Flight performance of actively foraging honey bees is reduced by a common pathogen. Environ. Microbiol. Rep..

[57] Grozinger CM, Flenniken ML (2019). Bee viruses: Ecology, pathogenicity, and impacts. Annu. Rev. Entomol..

[58] Zioni N, Soroker V, Chejanovsky N (2011). Replication of Varroa destructor virus 1 (VDV-1) and a Varroa destructor virus 1–deformed wing virus recombinant (VDV-1–DWV) in the head of the honey bee. Virol..

[59] Cornman RS (2017). Relative abundance of deformed wing virus, Varroa destructor virus 1, and their recombinants in honey bees (*Apis mellifera*) assessed by kmer analysis of public RNA-Seq data. J. Invertebr. Pathol..

[60] Campbell EM, Budge GE, Watkins M, Bowman AS (2016). Transcriptome analysis of the synganglion from the honey bee mite, Varroa destructor and RNAi knockdown of neural peptide targets. Insect Biochem. Mol. Biol..

[61] Giuffre C, Lubkin SR, Tarpy DR (2019). Does viral load alter behavior of the bee parasite *Varroa destructor*?. Plos one.

[62] Stich M, Briones C, Manrubia SC (2007). Collective properties of evolving molecular quasispecies. BMC Evol. Biol..

[63] Brutscher LM, Daughenbaugh KF, Flenniken ML (2015). Antiviral defense mechanisms in honey bees. Curr. Opin. Insect Sci..

[64] Roth O, Beemelmanns A, Barribeau SM, Sadd BM (2018). Recent advances in vertebrate and invertebrate transgenerational immunity in the light of ecology and evolution. Heredity.

[65] DeGrandi-Hoffman G, Chen YP (2015). Nutrition, immunity and viral infections in honey bees. Curr. Opin. Insect Sci..

[66] Annoscia D (2017). Elucidating the mechanisms underlying the beneficial health effects of dietary pollen on honey bees (*Apis mellifera*) infested by Varroa mite ectoparasites. Sci. Rep..

[67] Carrillo-Tripp J (2016). *In vivo* and *in vitro* infection dynamics of honey bee viruses. Sci. Rep..

[68] Ryabov EV, Fannon JM, Moore JD, Wood GR, Evans DJ (2016). The Iflaviruses Sacbrood virus and Deformed wing virus evoke different transcriptional responses in the honeybee which may facilitate their horizontal or vertical transmission. PeerJ.

[69] Jerzak GVS, Bernard K, Kramer LD, Shi P-Y, Ebel GD (2007). The West Nile virus mutant spectrum is host-dependent and a determinant of mortality in mice. Virology.

[70] Cabot B (2000). Nucleotide and amino acid complexity of Hepatitis C virus quasispecies in serum and liver. J. Virol..

[71] Laskus T, Radkowski M, Wang L-F, Nowicki M, Rakela J (2000). Uneven distribution of Hepatitis C virus quasispecies in tissues from subjects with end-stage liver disease: confounding effect of viral adsorption and mounting evidence for the presence of low-level extrahepatic replication. J. Virol..

[72] Forton DM, Karayiannis P, Mahmud N, Taylor-Robinson SD, Thomas HC (2004). Identification of unique Hepatitis C virus quasispecies in the central nervous system and comparative analysis of internal translational efficiency of brain, liver, and serum variants. J. Virol..

[73] Sutton TC (2014). Airborne Transmission of Highly Pathogenic H7N1 Influenza Virus in Ferrets. J. Virol..

[74] Sang X (2015). Adaptation of H9N2 AIV in guinea pigs enables efficient transmission by direct contact and inefficient transmission by respiratory droplets. Sci. Rep..

[75] Fürst MA, McMahon DP, Osborne JL, Paxton RJ, Brown MJF (2014). Disease associations between honeybees and bumblebees as a threat to wild pollinators. Nature.

[76] Martin SJ, Brettell LE (2019). Deformed wing virus in honeybees and other insects. Ann. Rev. Virol..

[77] Roossinck MJ (2015). Move over, bacteria! Viruses make their mark as mutualistic microbial symbionts. J. Virol..

[78] Tehel A, Brown MJF, Paxton RJ (2016). Impact of managed honey bee viruses on wild bees. Curr. Opin. Virol..

[79] Shapiro JW, Turner PE (2018). Evolution of mutualism from parasitism in experimental virus populations. Evolution.

[80] Evans, J. D. *et al*. Standard methodologies for molecular research in *Apis mellifera*. In: *The COLOSS BEEBOOK, Volume I: standard methods for Apis mellifera research* (Dietemann, V., Ellis, J. D. & Neumann, P. Eds.) Chapter 14. IBRA, Treforest, UK (2013).

[81] Thaduri S, Locke B, Granberg F, de Miranda JR (2018). Temporal changes in the viromes of Swedish varroa-resistant and varroa-susceptible honeybee populations. PLoS ONE.

[82] Li H (2009). The Sequence alignment/map (SAM) format and SAMtools. Bioinformatics.

[83] Acevedo A, Brodsky L, Andino R (2014). Mutational and fitness landscapes of an RNA virus revealed through population sequencing. Nature.

[84] Kumar S, Stecher G, Li M, Knyaz C, Tamura K (2018). MEGA X: Molecular Evolutionary Genetics Analysis across computing platforms. Mol. Biol. Evol..

[85] Librado P, Rozas J (2009). DnaSP v5: a software for comprehensive analysis of DNA polymorphism data. Bioinformatics.

[86] Nei, M. & Kumar, S. *Molecular evolution and phylogenetics*. Oxford University Press, Oxford, UK (2000).

[87] Tajima F (1989). Statistical method for testing the neutral mutation hypothesis by DNA polymorphism. Genetics.

[88] Fu YX, Li WH (1993). Statistical tests of neutrality of mutations. Genetics.

[89] Nei M, Gojobori T (1986). Simple methods for estimating the numbers of synonymous and nonsynonymous nucleotide substitutions. Mol. Biol. Evol..

[90] Jombart T (2008). Adegenet: An R package for the multivariate analysis of genetic markers. Bioinfo..

[91] Jombart T, Devillard S, Balloux F (2010). Discriminant analysis of principal components: a new method for the analysis of genetically structured populations. BMC Genetics.

[92] Liu L (2012). Comparison of Next-Generation Sequencing systems. J. Biomed. Biotech..

[93] Felsenstein, J. PHYLIP (Phylogeny Inference Package) version 3.65c. Distributed by the author. Department of Genetics, University of Washington, Seattle (1993).

[94] Lanzi G (2006). Molecular and biological characterization of deformed wing virus of honeybees (*Apis mellifera* L). J. Virol..

[95] Lamp B (2016). Construction and rescue of a molecular clone of Deformed wing virus (DWV). PLoS One.

[96] Barriga GP (2012). First detection and complete genome sequence of Deformed wing virus in Chilean honeybees. Virus Genes.

[97] Fujiyuki T (2004). Novel insect picorna-like virus identified in the brains of aggressive worker honeybees. J. Virol..

[98] Reddy KE (2013). Molecular characterization and phylogenetic analysis of deformed wing viruses isolated from South Korea. Vet. Microbiol..

[99] Forsgren E, de Miranda JR, Isaksson M, Wei S, Fries I (2009). Deformed wing virus associated with *Tropilaelaps mercedesae* infesting European honey bees (*Apis mellifera*). Exp. Appl. Acarol..

[100] Fei D (2019). Phylogenetic and recombination analyses of two deformed wing virus strains from different honeybee species in China. Peer J..

[101] Tamura K, Nei M (1993). Estimation of the number of nucleotide substitutions in the control region of mitochondrial DNA in humans and chimpanzees. Mol. Biol. Evol..

