# Supplementary material for: The honeybee (Apis mellifera) developmental state shapes the genetic composition of the deformed wing virus-A quasispecies during serial transmission
Source: Sci Rep. 2020 Apr 6;10:5956. doi: 10.1038/s41598-020-62673-w (PMC7136270; doi:10.1038/s41598-020-62673-w)
Supplement: Supplementary file 1 — Supplementary Figures Tables. [file 41598_2020_62673_MOESM1_ESM.pdf]

The honeybee (*Apis mellifera*) developmental state shapes the genetic composition of the deformed wing virus-A quasispecies during serial transmission

Orlando Yañez[1], Julio Chávez-Galarza[2,3], Christian Tellgren-Roth[4], M. Alice Pinto[2], Peter Neumann[1], Joachim R. de Miranda[5*]

Supplementary Files

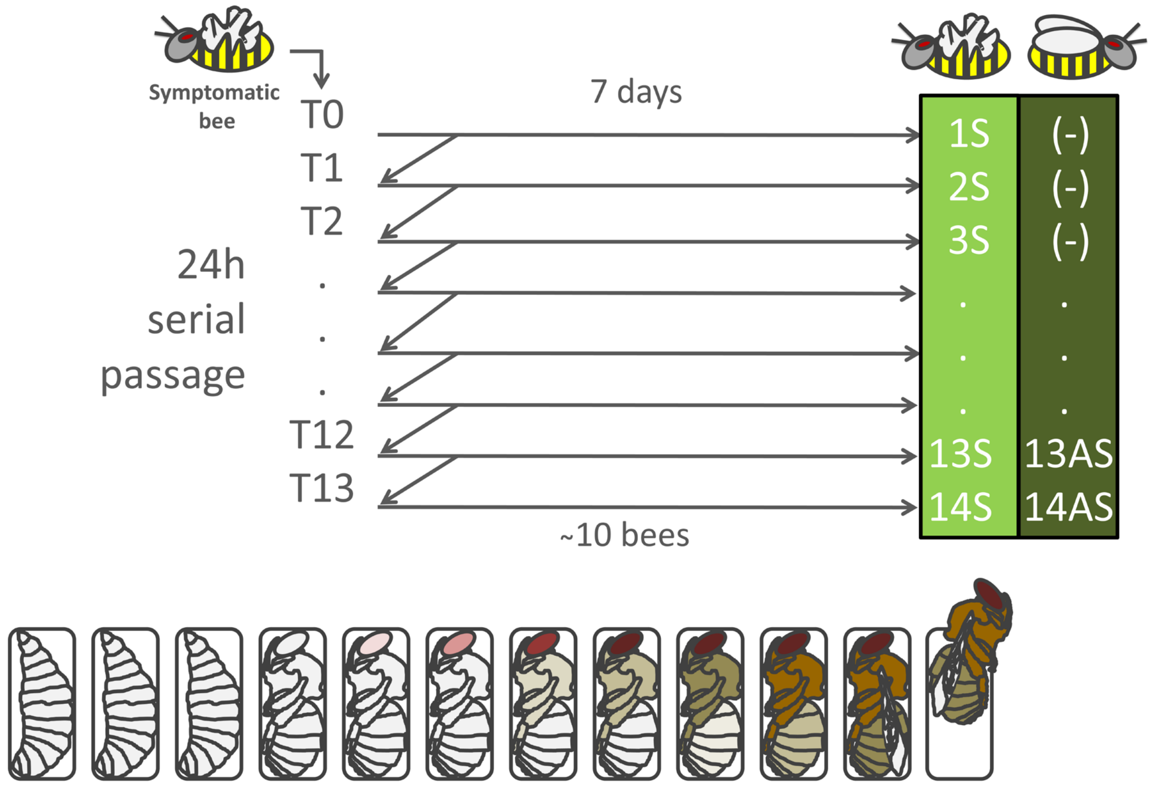

Supplementary Figure S1 | Serial transmission design. Experimental design outlining the organization of the serial transmission experiments relative to the bee pupal developmental phase (bottom), inoculating at the pink-eye stage, removing one pupa 24 hours later for preparing the inoculum for the next serial transfer and leaving the remaining pupae to complete development into either symptomatic (S; light green) or asymptomatic (AS; dark green) adults. T1-T13 refer to the number of the serial transfers from the original inoculum (T0).

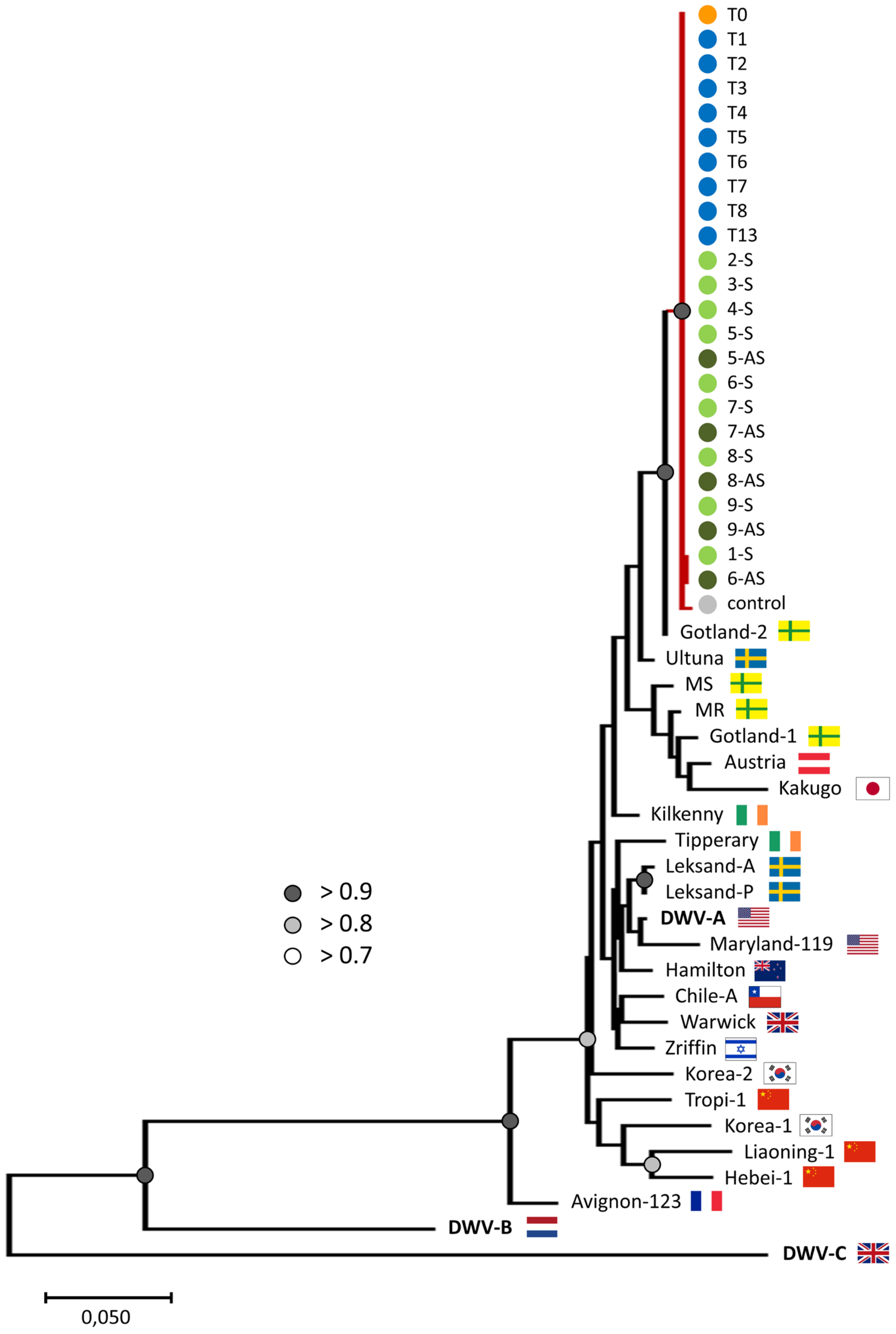

Supplementary Figure S2 | DWV phylogenetic relationships VIVA isolates

Reconstruction of the phylogenetic relationships between the DWV isolates of the current study to other Swedish isolates and a selection of international biogeographic isolates, based on the nucleotide sequence of the DWV Lp gene[14]. The relationships were inferred using the Maximum Likelihood method and the Tamura-Nei model of evolution[101], as implemented by MEGA-X[83]. The tree with the highest log likelihood (-3998,32) is shown. The statistical confidence of each of the nodes was determined by bootstrap analysis involving 500 replicates. Nodes with >70%, >80% and >90% probability are represented by open, shaded and closed circles respectively. Nodes with <70% probability are unmarked. The tree is drawn to scale, with branch lengths measured in the number of substitutions per site. The geographic origins of the isolates are indicated by flags. The accession number, geographic origin, year of isolation and original reference of the sequences used in the analyses are given in Supplementary Table 2.

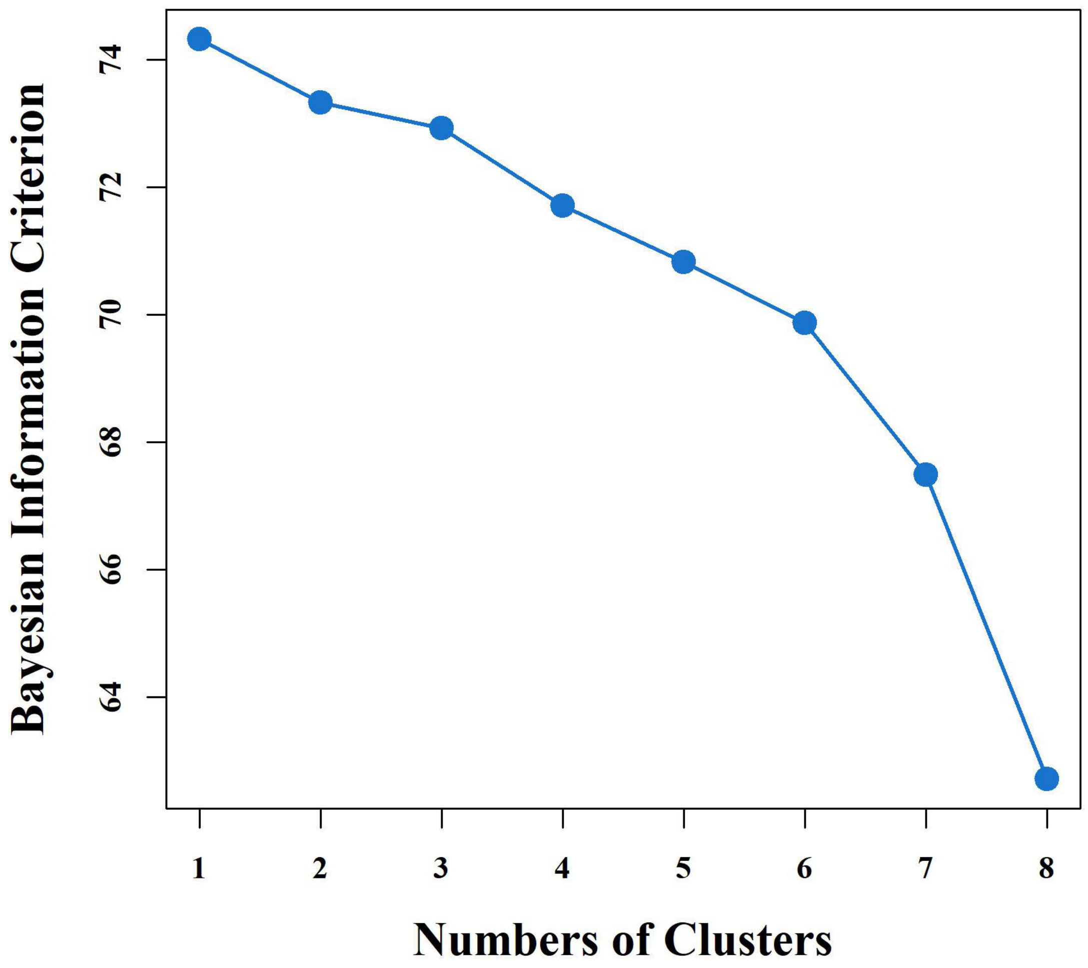

Supplementary Figure S3 | K-means genetic clusters determination. Optimum number of K-means genetic clusters among different groups of DWV quasispecies, as determined by Bayesian Information Criteria (BIC), for the serial transmission inocula presented in Figure 3D.

Purpose	Target[ref]	Primers	Sequence (5' to 3')	Size (nt)
Background virus analyses	DWV[23]	DWV-F8668	TTCATTAAAGCCACCTGGAACATC	136
		DWV-B8757	TTTCCTCATTAACTGTGTCGTTGA	
	ABPV[23]	ABPV-F6548	TCATACCTGCCGATCAAG	197
		KIABPV-B6707	CTGAATAATACTGTGCGTATC	
	BQCV[23]	BQCV-qF7893	AGTGGCGGAGATGTATGC	294
		BQCV-qB8150	GGAGGTGAAGTGGCTATATC	
	IAPV[23]	IAPV-F6627	CCATGCCTGGCGATTCAC	203
		KIABPV-B6707	CTGAATAATACTGTGCGTATC	
	SBV[23]	SBV-qF3164	TTGGAACTACGCATTCTCTG	335
		SBV-qB3461	GCTCTAACCTCGCATCAAC	
	SBPV[23]	SBPV-F3177	GCGCTTTAGTTCAATTGCC	226
		SBPV-B3363	ATTATAGGACGTGAAAATATAC	
	VDV-1[23]	VDV-F1409	GCCCTGTTCAAGAACATG	413
		DWV-B1806	CTTTTCTAATTCAACTTCACC	
	β-actin-iso2[23]	Am-actin2-qF	CGTGCCGATAGTATTCTTG	271
		Am-actin2-qB	CTTCGTCACCAACATAGG	
Whole genome amplification	DWV	DWV-F27	CATAGCGAATTACGGTGC	1817
		DWV-B1806	CTTTTCTAATTCAACTTCACC	
	DWV	DWV-F1725	GATTACGAGTTAGAGTGTG	1407
		DWV-B3095	GCTATTACTTTCCTGTAAATC	
	DWV	DWV-F3018	GTAGGTTAATTGTAGGTTATG	4317
		DWV-B7295	ACGCAGTTATACCAATTATAC	
	DWV	DWV-F7243	CCCGTGAAAATGATTCGTG	2897
		DWV-B10101	ACTATACTAAAATTAGGACGC	
Supplementary genome amplification	DWV[37]	DWV-F1153	ATTAAAAATGGCCTTTAGTTG	694
		DWV-B1806	CTTTTCTAATTCAACTTCACC	
	DWV	DWV-F3018	GTAGGTTAATTGTAGGTTATG	1355
		DWV-B4329	CAATTCTATAACATTATTTACACG	
	DWV	DWV-F4220	TTTGGGTACAACATCGACC	1485
		DWV-B5668	CACACTGATCCCAATAATC	
	DWV	DWV-F5625	ACGTGCGAGTCGTACTC	1706
		DWV-B7295	ACGCAGTTATACCAATTATAC	
	DWV	DWV-F7243	CCCGTGAAAATGATTCGTG	1588
		DWV-B8794	CCGTGAATATAGTGTGAGG	
	DWV	DWV-F8688	GGTAAGCGATGGTTGTTTG	1451
		DWV-B10101	ACTATACTAAAATTAGGACGC	

Supplementary Table S1. PCR primers used for virus quantification, DWV genome amplification and DI genome analyses. Shown are the purpose of the assay, the target virus (and the reference for the assay), the primer name and sequences and the size of the amplicon. Except where indicated, the assays were developed during this work.

Isolate[ref]	Accession	Year	Region
Leksand-P[37]	JF346656	2008	Dalarna, Sweden
Leksand-A[37]	JF346657	2008	Dalarna, Sweden
Ultuna[14]	JF346553	2006	Uppland, Sweden
Gotland-1[14]	JF346577	2006	Gotland, Sweden
Gotland-2[14]	JF346558	2006	Gotland, Sweden
MR[93]	MH267695	2009	Gotland, Sweden
MS[93]	MH267696	2009	Gotland, Sweden
Austria[95]	KU847397	2012	Vienna, Austria
Kakugo[97]	AB070959	1999	Chiba, Japan
Tipperary[14]	JF346636	2007	Tipperary, Ireland
Kilkenny[14]	JF346635	2007	Kilkenny, Ireland
Maryland-119[34]	MH069506	2015	Maryland, USA
Hamilton[26]	MF623172	2015	Hamilton, New Zealand
Chile-A[96]	JQ413340	2011	Chile, South America
Warwick[20]	GU109335	2009	Warwickshire, England
Zriffin[58]	JF440526	2008	Bet Dagan, Israel
Korea-1[98]	JX878304	2012	Gyeonggi, South Korea
Korea-2[98]	JX878305	2012	Gyeonggi, South Korea
Tropi-1[99]	JF346640	2007	Hainan, China
Liaoning-1[100]	MF770715	2017	Liaoning, China
Hebei-1[100]	MH165180	2018	Hebei, China
Avignon-123[32]	KX373900	2013	Avignon, France
DWV-A[94]	AY292384	2000	Pennsylvania, USA
DWV-B[48]	AY251269	2001	Gelderland, The Netherlands
DWV-C[33]	CEND01000001	2006	Devon, England

Supplementary Table S2. Sequences used in the phylogenetic analyses of Swedish DWV isolates. Shown are the name of the isolate, including the original reference, the accession number, the year of isolation and the region where the isolate was found.
